# Synovial Immunohistological Biomarkers of the Classification of Undifferentiated Arthritis Evolving to Rheumatoid or Psoriatic Arthritis

**DOI:** 10.3389/fmed.2021.656667

**Published:** 2021-04-09

**Authors:** Andrea Cuervo, Raquel Celis, Antonio Julià, Alicia Usategui, Regina Faré, Julio Ramírez, Ana Belen Azuaga, Andrés Lorenzo, Raimon Sanmartí, José L. Pablos, Juan D. Cañete

**Affiliations:** ^1^Arthritis Unit, Department of Rheumatology, Hospital Clínic, University of Barcelona and Institut d'Investigacions Biomèdiques August Pi I Sunyer (IDIBAPS), Barcelona, Spain; ^2^Rheumatology Research Group, Vall d'Hebron Research Institute, Barcelona, Spain; ^3^Department of Rheumatology, Research Institute Hospital 12 de Octubre, Complutense University of Madrid, Madrid, Spain; ^4^Rheumatology Division, Hospital Universitario de Burgos, Burgos, Spain

**Keywords:** synovitis, undifferentiated arthritis, biomarkers, rheumatoid arthritis, psoriatic arthritis

## Abstract

**Background:** Undifferentiated arthritis (UA) is defined as an inflammatory arthritis that does not fulfill criteria for a definite diagnosis. Delay in reaching a specific diagnostic and therapy may lead to impaired functional outcomes. Our aim was to identify synovial biomarkers associated with definitive diagnostic classification in patients with UA.

**Methods:** DMARD-naïve UA patients with available initial synovial tissue (ST) and a final diagnosis of rheumatoid arthritis (RA) or psoriatic arthritis (PsA) during follow-up were included and compared with patients with well-defined disease (RA or PsA). Clinical, arthroscopic, and pathological data were compared between groups. Pathology included quantitative immunohistochemical (IHC) analysis of cell types and human interferon-regulated MxA. Principal component analysis (PCA) was performed to extract disease patterns.

**Results:** One hundred and five patients were included: 31 patients with DMARD-naïve UA (19 evolving to RA and 12 to PsA during a median follow up of 7 years), 39 with established RA, and 35 with established PsA. ST from the UA group showed higher macrophage density compared with the established RA and PsA groups. Patients with UA evolving to RA (UA-RA) showed higher MxA expression and CD3^+^ T-cell density compared with established RA. UA patients evolving to PsA (UA-PsA) showed increased vascularity and lining synovial fibroblast density compared with established PsA. Synovitis of UA-PsA patients showed more mast cells and lining fibroblasts compared with UA-RA. No between-group differences in local or systemic inflammation markers were found.

**Conclusions:** Our results show differences in the cellular composition of UA synovium compared with RA and PsA, with higher density of the cellular infiltrate in the UA groups. Initial expression of the interferon inducible gene MxA could be a biomarker of progression to RA, while higher mast cell and fibroblastic density may be associated with PsA progression.

## Introduction

Undifferentiated arthritis (UA) is defined as an inflammatory arthritis not satisfying classification criteria for rheumatoid arthritis (RA) (1–3) or peripheral spondyloarthritis (pSpA) (4), including psoriatic arthritis (PsA) (5). UA represents 33–35% of patients in early arthritis clinics, usually as mono- or oligo-arthritis. The UA course is variable (mild, chronic, or self-limited disease), and ~20–50% of patients do not fulfill definite disease classification criteria after 1 year of follow up (6–8). In our early arthritis cohort (*n* = 381), we found that 30% of patients met criteria for RA, 10% for SpA and 16% did not fulfill criteria for any specific disease after 1 year of follow up (6).

The differential diagnosis between RA and pSpA in the earliest stages may be difficult due to the lack of specific classification criteria. The identification of patients with UA who will progress to RA or PsA might be helpful to tailor early therapy and prevent overtreatment. Delay in reaching a definitive diagnosis and appropriate therapy may lead to persistent joint inflammation and damage and poor functional outcomes.

Macroscopic synovial features and immunohistochemical (IHC) analysis of synovial membrane may be useful in evaluating patients with UA. Arthroscopic assessment of the synovial vascular pattern suggests the straight pattern is more specific of early RA, while a tortuous pattern points to early SpA (9, 10). Studies have compared the synovial histopathology of RA and pSpA, including PsA. Sublining CD68^+^ macrophage density and ectopic lymphoid neogenesis (ELN) are equally present in PsA and RA. However, RA is characterized by greater lining hyperplasia and PsA by a greater vessel, neutrophil, mast cell and CD163^+^ macrophage density (11, 12).

Increased expression of type I interferon inducible genes (interferon gene signature or IGS) has been variably described in RA and PsA tissues. Interestingly, in RA it is more prominent in the early or pre-arthritic phases where it is potentially predictive of disease progression and worse responses to therapy. Although the specific contribution of the induced genes to the pathogenesis of arthritis is not known, multiple effects on innate and adaptive immune responses in other autoimmune diseases have been described (13–15). The aim of this study was to analyze synovial tissue and vascular patterns as potentially-useful predictive biomarkers of the transition from undifferentiated to definite arthritis.

## Materials and Methods

### Patients and Synovial Tissue

We made a retrospective case-control study comparing a cohort of active DMARD-naïve UA patients with patients with active definitive disease (39 with established RA and 35 with established PsA) collected by the Arthritis Unit, Hospital Clinic, Barcelona, between 2000 and 2014. We selected all patients who fulfilled the classification criteria for RA {1987 ACR (2) or PsA [CASPAR criteria (5)]} during the follow-up and had synovial tissue obtained by diagnostic and therapeutic (lavage) needle arthroscopy of the knee at the time they had UA. Thirty-one DMARD-naïve UA patients were included. Nineteen patients (UA-RA) met classification criteria for RA and 12 (UA-PsA) for PsA. Clinical and demographic characteristics are shown in Table 1. UA patients predominantly had oligo- and polyarticular disease and moderate disease activity. Five patients had received prednisone (<7.5 mg/day).

**Table 1:** Demographic, clinical, and serological data.

***n* = 105**	**UA**	**UA-RA**	**RA**	***p***	**UA-PsA**	**PsA**	***P***
	***n* = 31**	***n* = 19**	***n* = 39**		***n* = 12**	***n* = 35**	
Age (years)	48 ± 13	48 ± 13	59 ± 12	**0.012**	48 ± 14	53 ± 13	0.244
Sex (male) *n* (%)	14 (45)	5 (26)	16 (41)	0.385	9 (75)	22 (63)	0.505
Disease duration (months)	24 ± 24 (2–85)	30 ± 28 (3–85)	40 ± 70 (1–304)	0.765	16 ± 12 (2–83)	22 ± 24 (3–99)	0.625
SJC	3.10 ± 3.04 (1–14)	4.21 ± 3.44 (1–14)	6.72 ± 6.30 (1–28)	0.204	1.33 ± 0.65 (1–3)	2.31 ± 2.18 (0–9)	0.159
TJC	4.39 ± 4.94 (1–17)	6.21 ± 5.60 (1–17)	6.95 ± 7.87 (0–28)	0.802	1.50 ± 0.67 (1–3)	2.37 ± 2.49 (1–11)	0.413
CRP basal (mg/dl)	2.47 ± 2.24	2.67 ± 2.02	3.92 ± 3.23	0.226	2.14 ± 2.60	3.17 ± 3.95	0.483
ESR basal (mm/h)	37 ± 27	41 ± 25	46 ± 31	0.785	29 ± 30	36 ± 35	0.677
DAS28 basal (ESR)	4.24 ± 1.07	4.75 ± 0.91	4.84 ± 1.42	0.892	3.43 ± 0.79	3.76 ± 1.03	0.239
RF *n* (%)	5 (16)	5 (26)	26 (67)	**0.034**	0	0	-
ACPA *n* (%)	4 (13)	4 (21)	31 (80)	**0.016**	0	0	-
HLA B27 *n* (%)	2 (7)	0	0	-	2	11 (31)	0.461
**Articular pattern**						
Monoarthitis	7 (23)	3 (16)	0	**0.001**	4 (33)	3 (9)	**0.038**
Oligoarthritis	10 (32)	3 (16)	0		7 (58)	18 (51)	
Polyarthritis	14 (45)	13 (68)	39 (100)		1 (8)	14 (40)	
csDMARD *n* (%)	0	0	22 (56)	**-**	0	13 (37)	**-**
bDMARD *n* (%)	0	0	8 (21)	**-**	0	5 (14)	**-**

*Data are expressed as mean ± SD (IQR)*.

*UA, undifferentiated arthritis; RA, rheumatoid arthritis; PsA, psoriatic arthritis; UA-RA, UA evolving to R; UA-PsA, UA evolving to PsA; SJC, swollen joint count; TJC, tender joint count; CRP, C-Reactive Protein; ESR, erythrocyte sedimentation rate; DAS, disease activity score; RF, rheumatoid factor, ACPA, anti-citrullinated protein antibody; csDMARD, conventional synthetic disease-modifying antirheumatic drug; bDMARD, biologic disease-modifying antirheumatic drug (anti TNF-alpha therapy)*.

*p-value in bold remarks statistical significance*.

Synovial biopsy specimens were formalin fixed and paraffin embedded for immunohistochemistry. The study was approved by the institutional ethics committee of the Hospital Clinic, Barcelona, Spain (HCB/2014/0579). All patients provided written informed consent.

Clinical data from the four groups were recorded: age, sex, time of follow up until meeting classification criteria, number of swollen and tender joints, CRP, ESR, disease activity score by ESR (DAS28-ESR), rheumatoid factor and/or ACPA status, HLA-B27 status, and treatment at time of arthroscopy (NSAIDs, glucocorticoids, csDMARDs, bDMARDs).

All arthroscopy records were reviewed by JDC and AC to analyze the macroscopic characteristics of synovitis: vascular synovial pattern (straight, tortuous, or mixed) (9, 10).

### Immunohistochemistry

Synovial biomarkers were analyzed by IHC. Biopsies were stained with the following antibodies: anti-CD3 (T cells, polyclonal rabbit anti-human CD, clone A045, DAKO, Cambridge, UK), anti-CD20 (B cells, mouse anti-human CD20:clone L26, DAKO), anti-CD138 (plasma cells, clone B-B4; Santa Cruz Biotechnology, Inc., San Diego, CA, USA), anti-CD68 (macrophages, IgG1 KP1 clone; Dako), anti-CD117 (mast cells, rabbit anti-human polyclonal antibody; Dako), anti-CD15 (neutrophils, clone BY87; Novocastra), anti-CD31 (endothelial cells, clone JC70A), anti-hsp47 (synovial fibroblasts, IgG2b clone M16.10A1; Assay Designs), anti-PDC (6W-DEN-DDX0043P, monoclonal antibody to C.D303. IgG1 clone 124B3.13). To obtain quantitative data on the expression of an type I IFN-inducible factor in ST, we selected the antiviral response protein MxA that has provided a robust readout of type I IFN response in tissues from other autoimmune conditions [rabbit polyclonal anti-MX1 of Abcam (Ref: ab95926)] (16, 17). Parallel sections were incubated with irrelevant isotype- and concentration-matched monoclonal antibodies as negative controls. Sections were finally counterstained in Gill's hematoxylin.

ST cell infiltration was assessed using CD3 (T cells), CD20 (B cells), CD79 (pre-plasma cells), CD138 (plasma cells), CD31 (vessels), CD68 (macrophages: lining and sublining), CD15 (neutrophils), CD117 (mast cells), Hsp47 (fibroblasts: lining and sublining); anti-PDC (plasma dendritic cells) and ectopic lymphoid neogenesis (ELN) in the UA samples were compared with the respective control groups. The entire area of each tissue specimen was photographed and digitized using a SPOT RT CCD camera and SPOT 4.0.4 software (Diagnostic Instruments) on an Axioplan 2 fluorescence microscope (Zeiss). For the quantitative evaluation (cells/mm^2^), tissues were sequentially photographed using Digital Image Analysis (Olympus). The highest grade of lymphoid aggregation within each sample was determined according to a previously described scoring method (18, 19) based on the number of radial cell counts: grade 1 = 2–5 radial cell counts, grade 2 = 6–10 radial cell counts, and grade 3 ≥ 10 radial cell counts. ELN was defined histologically as follicular aggregates grade ≥ 2 with T/B cell segregation.

### Statistical Analysis

Data were analyzed using the SPSS 18.0 statistical program (SPSS, Chicago, IL). Fisher's exact-test was used to evaluate associations between the presence or absence of all qualitative variables. Between-group quantitative variables were compared using ANOVA. Violin and box plots were generated in R software (www.R-project.org) with functions from the *ggplot2* package (doi: 10.1007/978-0-387-98141-3). Spearman's rank correlation was used for associations. *P*-values < 0.05 were considered statistically significant.

Principal component analysis (PCA), a multidimensional analysis technique that can extract patterns from large numbers of variables, was used as a multivariate technique (20) to examine variations in the marker data set and whether any clustering was presented in relation to the study groups. We selected only variables with no missing values or 1 missing value (which was imputed). PCA was performed for: T lymphocytes (anti-CD3), neutrophils (anti-CD15), B cells (anti-CD20), endothelial cells (anti-CD31), macrophages (anti-CD68), pre-B cells (anti-CD79), mast cells (anti-CD117), and plasma cells (anti-CD138).

## Results

### Clinical and Demographic Characteristics

Thirty-one DMARD-naïve UA patients were included. Nineteen patients (UA-RA) met 1987 ACR classification criteria for RA (including 4 patients that also fulfill 2010 ACR criteria) and 12 for PsA. Synovial tissue from 39 patients with active established RA and 35 with active established PsA were also included. Clinical and demographic details are shown in Table 1.

Regarding the pharmacological treatment used during the long follow-up in UA patients, UA patients evolving to RA received more csDMARDs (89% were treated with a mean of 1.5 cDMARDs and currently 71% of them were taken methotrexate and 29% leflunomide) and bDMARDs [4 (21%) patients had been treated with a mean of 1.5 biologics, and currently 6 have biologics treatment−2 etanercept, 2 tocilizumab, and 2 abatacept-] than UA patients evolving to PsA (33% of patients had been treated with methotrexate, and currently 2 were taken methotrexate and 2 others patients adalimumab). These findings are expected as UA-PsA had more mono-oligoarthritis and the disease is more localized and intermittent.

### Arthroscopic Vascular Patterns

The most common pattern in UA patients was a predominantly tortuous pattern, regardless of classification to RA or PsA: 84% (16/19) of UA-RA patients and 100% (12/12) UA-PsA. The remaining UA-RA patients showed a mixed (2 patients) or straight (1 patient) pattern. The vascular patterns were 58% (21/36) tortuous, 31% (11/36) mixed, and 11% (4/36) straight in the definitive RA group and 88% (30/34) tortuous pattern, 9% (3/34) mixed, and 3% (1/34) straight pattern in the definite PsA group. No significant between-group differences were found in the distribution of the vascular patterns, although most of the few patients with a straight pattern had UA-RA or definitive RA.

### Quantitative IHC Cellular Patterns

**Figure 1:**
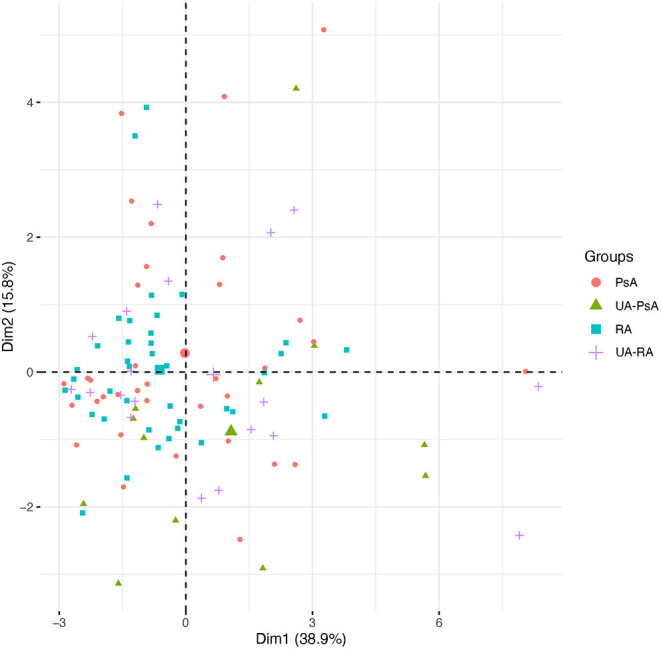
Principal Component analysis (PCA). Distribution of samples according to the PC1 and PC2. The variables included were those that had no missing values or those with as maximum 1 missing value (which we impute): CD3^+^ T-cells, CD20^+^ B-cells, CD79^+^ B-cells, CD138^+^ plasma-cells, CD31^+^ endothelial cells, CD68^+^ lining macrophages, CD68^+^ sublining, CD79^+^ B-cells, CD15^+^ neutrophils, and CD117^+^ mast cells. Reducing the data dimensionality to the main PCs does not show a clear separation between any of the 4 groups of patients.

PCA analyses of immunohistological markers showed no clear separation between groups (Figure 1).

No differences in individual markers between established RA and PsA were found. We then compared the distribution of all markers between UA-RA and UA-PsA and between UA and the definitive RA and PsA groups.

Overall, the UA group had a greater density of CD68^+^ sublining macrophages compared with the definitive RA and PsA groups (*p* = 0.016). No other differences were found.

**Figure 2:**
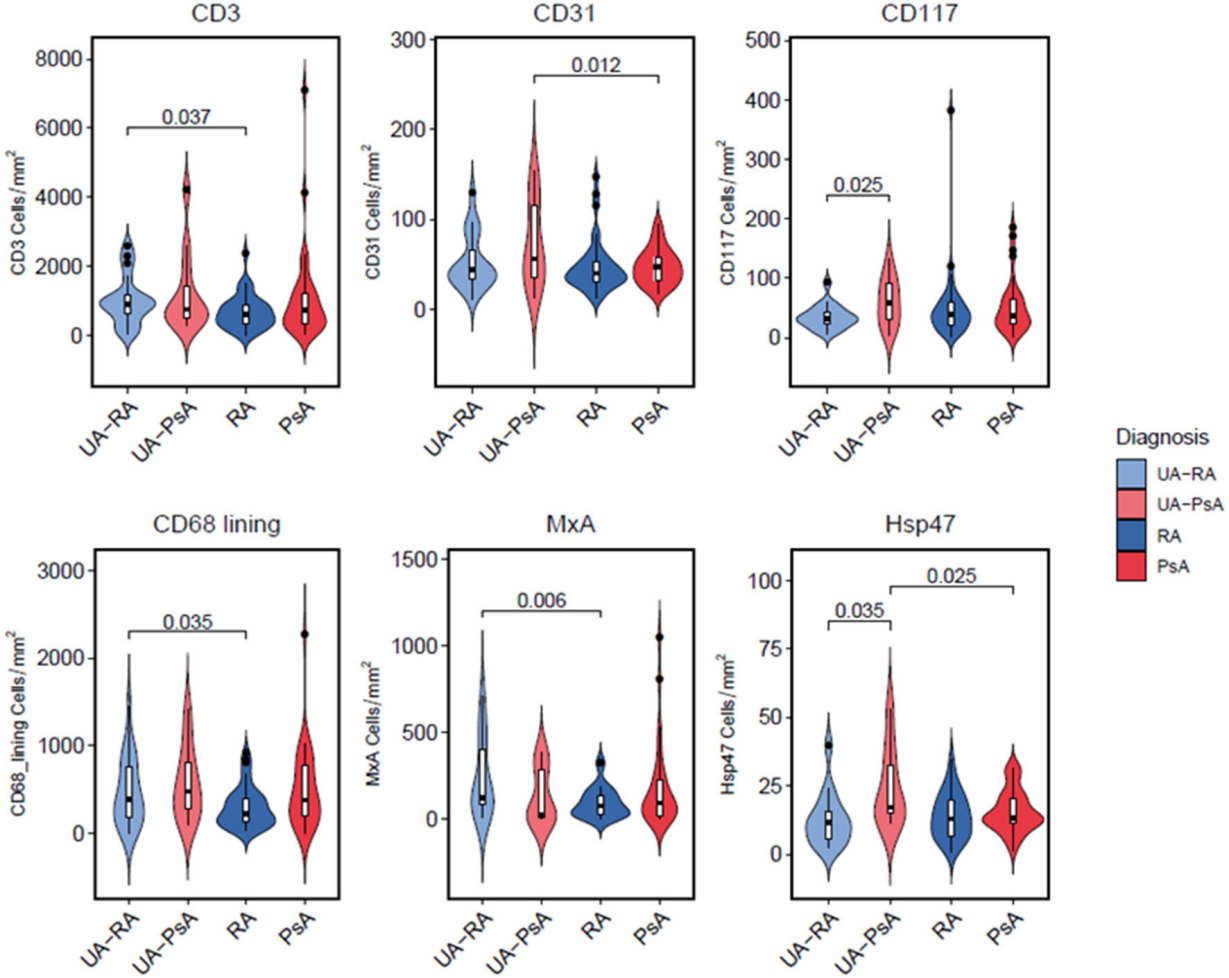
Characterization of cellular patterns by patient group. Violin plots depicting density distributions of positive cells (in area = 1 mm^2^), stained with surface protein antibodies and analyzed by immunohistochemitry. Inner boxplots show median, interquartile range, minimum and maximum values of every analyzed surface marker. Undifferentiated Arthitis (UA-RA, UA-PsA) and definite Rheumatoid Arthritis (RA) and Psoriatic Arthritis (PsA) using ANOVA-test, *p*-values between different groups are depicted for each marker.

Comparison between the UA-RA and definitive RA groups showed higher lining CD68^+^ macrophage and CD3^+^ T cell densities in the UA-RA group (*p* = 0.035 and *p* = 0.037, respectively) (Table 2 and Figure 2).

**Table 2:** Synovial tissue immunohistochemistry from undifferentiated arthritis, definite RA and definite PsA.

***n* = 105**	**UA**	**UA-RA**	**RA**	**UA-RA vs. RA**	**UA-PsA**	**PsA**	**UA-PsA vs. PsA**	**UA-RA vs. UA-PsA**
	***n* = 31**	***n* = 19**	***n* = 39**	***p-*value**	***n* = 12**	***n* = 35**	***p-*value**	***p-*value**
CD3	1,083	1,007	664	**0.037**	1,203	1,067	0.754	0.567
CD15	207	287	146	0.112	81	260	0.185	0.113
CD20	266	298	257	0.546	216	362	0.245	0.361
CD31	60	52	48	0.599	74	48	**0.012**	0.141
CD68L	512	477	300	**0.035**	568	502	0.653	0.544
CD68SL	1,835	1,769	1,121	0.112	1,939	1,115	0.068	0.801
CD79	707	817	558	0.271	540	536	0.987	0.505
CD117	45	35	50	0.286	60	53	0.625	**0.025**
CD138	682	743	749	0.982	585	1,001	0.423	0.639
%Hsp47 L^*^	17	13	14	0.656	25	16	**0.025**	**0.036**
%Hsp47 SL	5	5	6	0.593	5	4	0.239	0.951
MxA	189	242	97	**0.006**	132	187	0.495	0.202
antiPDC	79	73	61	0.579	88	98	0.797	0.609
ELN (%)	15 (48)	9 (47)	23 (59)	0.574	6 (50)	17 (49)	1.000	1.000

*Mean comparison*.

* *Data shows cells number/mm^2^ and percentage*.

*UA, undifferentiated arthritis; RA, rheumatoid arthritis; PsA, psoriatic arthritis; UA-RA, UA evolving to RA; UA-PsA, UA evolving to PsA; L, lining; SL, sublining; HsP47, heat shock protein 47; MxA, antiviral response protein MxA; PDC, plasmocytoid dendritic cell; ELN, Ectopic lymphoid neogenesis. p-value in bold remarks statistical significance*.

Comparison between UA-PsA and definitive PsA showed higher CD31^+^ vessel and Hsp47^+^ lining synovial fibroblast densities (*p* = 0.012 and *p* = 0.025, respectively) in the UA-PsA group (Table 2 and Figure 2).

Differences between UA evolving to RA or PsA (UA-RA vs. UA-PsA) were only found in CD117^+^ mast cells and Hsp47^+^ lining synovial fibroblasts densities, which were both higher in the UA-PsA group (*p* = 0.025 and *p* = 0.036, respectively) (Table 2 and Figure 2).

**Figure 3:**
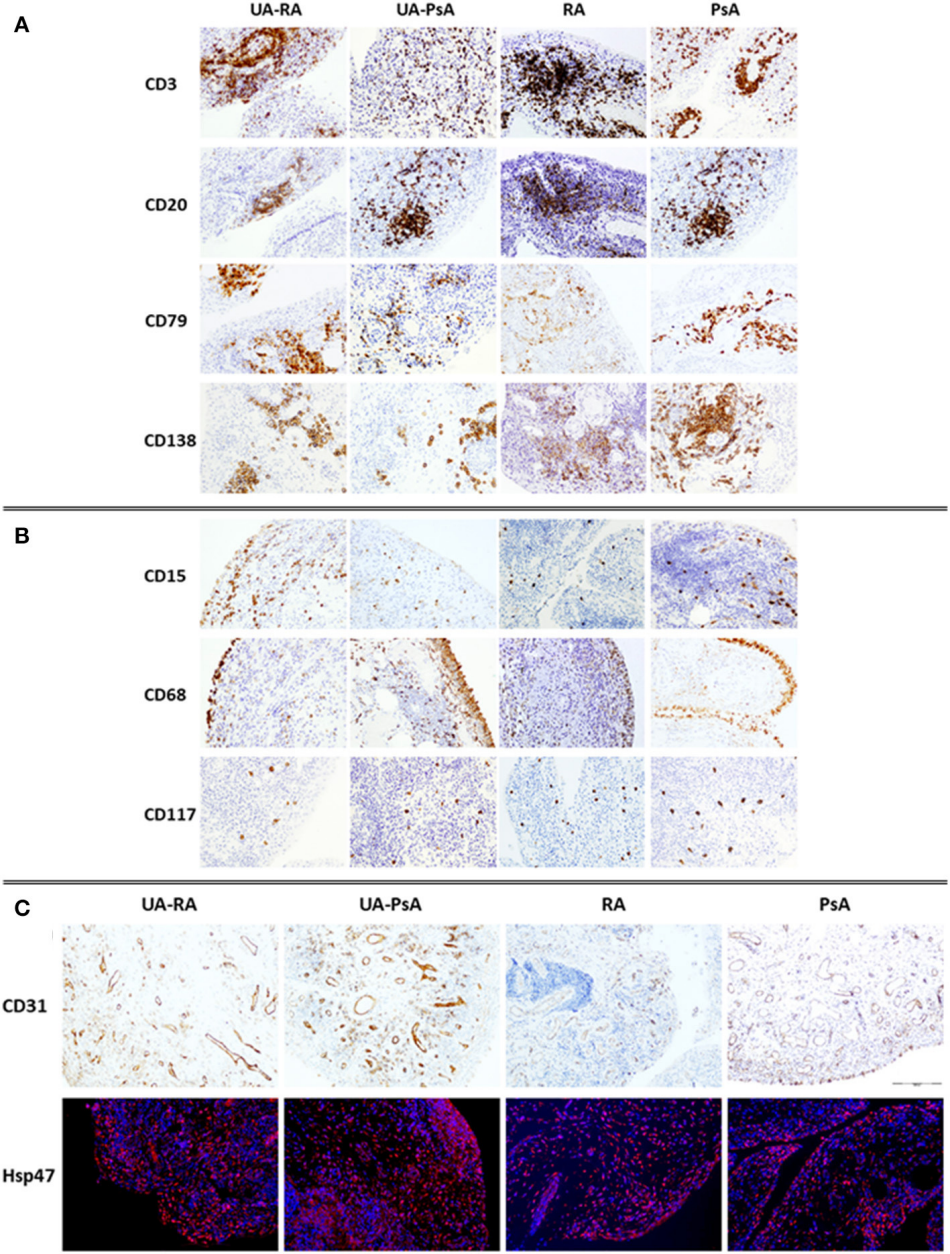
Immunostaining of cellular infiltrate in synovial membrane of UA-RA, UA-PsA, RA and PsA patients. **(A)** Adaptive immune cells (CD3, CD20, CD79, and CD138, 20 × ); **(B)** innate immune cells (CD68, CD15, and CD117, 20 × ); and **(C)** stroma cells (CD31, 10 × ) and Immuno-fluorescence labeling of fibroblast Hsp47^+^ (red) in sections from synovial tissue. DAPI (blue) nuclear counterstained; original magnification 20 ×. RA, rheumatoid arthritis; PsA, psoriatic arthritis; UA, undifferentiated arthritis evolving to RA (UA-RA) or PsA (UA-PsA).

No between-group differences in B cells, pre-plasma cells, plasma cells, neutrophils, plasmocytoid-dendritic cells or ELN were found (Figure 3). Also we do not found a significant impact of autoantibody positivity on synovial tissue IHC.

### MxA Expression in UA Evolving to RA or PsA

Analyses of MxA expression showed the UA group had non-significantly higher MxA expression compared with patients with definitive arthritis (189 vs. 145 cells/mm^2^, respectively). Definitive RA patients showed a trend to a significantly lower MxA expression than definitive PsA patients (97 vs. 187 cells/mm^2^, respectively, *p* = 0.08). UA-PsA patients also had a non-significantly lower expression of MxA expression than definitive PsA patients (132 vs. 187 cells/mm^2^; *p* = 0.5). However, UA-RA patients had significantly higher MxA expression than definitive RA patients (242 vs. 97/mm^2^, respectively, *p* = 0.006) (Table 2 and Figure 2).

## Discussion

To the best of our knowledge, no studies have analyzed synovial tissue biomarkers of the transition from DMARD-naïve UA patients to well-defined disease (RA or PsA). Taken together, our results show that the undifferentiated stage of RA and PsA tends to have globally higher synovial density of stromal cells, myeloid cells and lymphocytes than the established phases of RA and PsA. UA evolving to PsA showed more CD117^+^ mast cells and Hsp47^+^ lining synovial fibroblasts than UA evolving to RA, whereas UA evolving to RA showed significantly higher MxA (IGS) expression, and more CD68^+^ macrophages and CD3^+^ lymphocytes. These differences may be useful in the differential diagnosis. Specifically, the combination of ST MxA expression and mast cell density might be useful in classifying UA patients with a higher probability of evolving to RA or PsA, respectively.

With respect to macrophages, our results agree with previous reports (11), as we found no differences between RA and PsA. However, sublining macrophage density was higher in UA patients compared with the definite RA and PsA groups, although there were no differences in local or systemic inflammation markers. The lack of DMARD treatment in the UA group might have contributed to this observation. However, we have previously shown that UA patients evolving to RA or PsA, but not UA patients remaining as UA, had a significant increase in CD163^+^ macrophages compared with definitive PsA or RA patients, independently of csDMARD therapy (21). Furthermore, a study in 42 patients with untreated undifferentiated inflammatory seronegative arthritis reported that 6 patients who differentiated during the follow up (2 RA, 2 PsA, and 2 pSpA) had significantly higher synovial CD68^+^ macrophage scores compared with patients remaining undifferentiated, in line with our results (22).

Besides the higher density of CD3^+^ T cells, which has also been observed in UA evolving to definitive arthritis (22), we also found a high expression of the type 1 interferon gene signature (MxA) in patients with UA evolving to RA but not in those with PsA or definitive RA. The activation of this pathway may be one of the triggers involved in the initiation of early inflammatory events in patients with RA. One study found that MxA, IFI6, OAS1, ISG15, and IFI44L expression was significantly higher in patients with non-treated early RA than in those with established RA, and a significant and sustained decrease in IGS expression was found after 6 months of treatment (14). Therefore, this pathway could be a potential biomarker in UA patients evolving to RA (23).

In line with other studies, we found a low expression of pDC in UA-RA synovium, suggesting a different cellular origin of type 1 IFN activator of this pathway in early RA (24).

Analysis of the macroscopic vascular pattern of synovitis identified a predominantly tortuous pattern in UA patients, similar to that reported in early SpA (9). In the established PsA group, 88% of patients had a tortuous pattern, compared with 58% in established RA. Only 6 patients had a straight vascular pattern, of whom 5 had UA-RA or established RA. In previous studies, we found an association between the straight pattern and RF^+^ RA, whereas the tortuous pattern was associated with RF^−^ RA (10). Given the difficulty in qualitatively evaluating this feature, quantification of vascular patterns (vascular score, VS) has been made by giving 1 point for a straight pattern, 2 points for a mixed pattern and 3 point for a tortuous pattern (25). Despite the small sample size [7 RA (VS: 1.45) and 8 PsA (VS: 2.50)], the study suggests the existence of mixed patterns and the difficulty in assigning a diagnosis to a specific vascular pattern. Large, multicenter, prospective studies to clarify the diagnostic role of synovial vascular patterns in undifferentiated arthritis are needed.

PsA synovitis is characterized by prominent vascularization and an increased number of neutrophils, mast cells and CD163^+^ macrophages (11). We found a higher number of mast cells and lining fibroblasts in UA-PsA patients than in UA-RA patients. Mast cells have been reported to be specifically increased and containing higher levels of IL-17 in PsA than in RA synovitis, which may contribute to the progression of joint inflammation and damage (26). Mast cells capture and store exogenous IL-17A in intracellular granules through receptor-mediated endocytosis and release it after mast cell stimulation (27). A recent study in RA patients showed that high ST mast cells are associated with local and systemic inflammation, autoantibody positivity and high disease activity; interestingly, mast cells reside at the outer border of lymphoid aggregates and promote the activation and differentiation of naïve B-cells and induce the production of ACPA (28). Our results suggest that synovial mast cells are already increased in the undifferentiated phase of PsA, even at higher levels than that in established PsA. This finding could be useful to predict differentiation from UA to definitive PsA.

UA-PsA patients also had a higher density of vessels and lining fibroblast compared with established PsA patients. Synovial fibroblasts in PsA have been related to a greater angiogenic response in early PsA compared with early RA (25). However, no studies have compared synovial fibroblasts between the undifferentiated and established phases of PsA.

Our study has some limitations, such as the retrospective design, the heterogeneity of the cohort in terms of disease duration and treatment received, the different number of cs- and bDMARDs received by UA patients evolving to RA vs. PsA during the transition period, and the small number of patients in the UA-PsA group, which might have affected the results. Moreover, the UA group includes both early and late arthritis and therefore the UA patients cohort are not exactly an “early” arthritis cohort. Finally, as our UA cohort was recruited since year 2000, we applied the 1987 ACR criteria for classification of RA and, naturally, the results of our study do not can be applied to 2010 ACR criteria for RA. However, it is the first study to evaluate synovial tissues in DMARD-naïve UA evolving to established arthritis (RA, PsA) during a long-term follow-up, and provides some interesting clues that merit confirmation in larger cohorts.

## Conclusion

Our results highlight the predominant tortuous vascular pattern and increased CD68^+^ macrophage density in UA patients compared with patients with definitive RA or PsA. Increased expression of MxA in UA-RA could be a biomarker and suggests activation of the IGS pathway from the earliest phases of RA. In contrast, high mast cell density characterizes the undifferentiated phase of PsA and could be a biomarker of differentiation to definitive PsA. Finally, higher density of vessels and lining fibroblasts characterizes the undifferentiated phase of PsA compared with definitive PsA. These potential biomarkers of transition from the undifferentiated state to definitive arthritis requires validation in larger, prospective studies.

## Data Availability Statement

The original contributions generated for the study are included in the article/supplementary material, further inquiries can be directed to the corresponding author.

## Ethics Statement

The studies involving human participants were reviewed and approved by Ethics Research Committee of the Hospital Clinic of Barcelona, Spain (HCB/2014/0579). The patients/participants provided their written informed consent to participate in this study.

## Author Contributions

JC had full access to all the study in and takes responsibility for the integrity of the data and the accuracy of the data analysis and was responsible for the study design. AC, RC, AA, and JR performed clinical data acquisition. AJ, AC, AU, RF, and JP were responsible for the analysis. RC, AC, AU, RF, RS, JP, and JC performed data interpretation. Manuscript preparation was by AC, AJ, JP, and JC. All authors read and approved the final manuscript.

## Conflict of Interest

The authors declare that the research was conducted in the absence of any commercial or financial relationships that could be construed as a potential conflict of interest.
